# Intestinal Fatty Acid Binding Protein as a Predictor of Early Mesenteric Injury Preceding Clinical Presentation: A Case Report

**DOI:** 10.1016/j.ejvsvf.2024.04.004

**Published:** 2024-04-29

**Authors:** Annet A.M. Duivenvoorden, Flores M. Metz, Robin Wijenbergh, Hanne C.R. Verberght, Annemarie A.J.H.M. van Bijnen, Steven W.M. Olde Damink, Robert H. Geelkerken, Kaatje Lenaerts, Tim Lubbers

**Affiliations:** a Department of Surgery, NUTRIM School of Nutrition and Translational Research in Metabolism, Maastricht University, Maastricht, the Netherlands; b Department of Vascular Surgery, Medisch Spectrum Twente, Enschede, the Netherlands; c Multi-Modality Medical Imaging Group, TechMed Centre, University of Twente, Enschede, the Netherlands; d Department of Surgery, Maastricht University Medical Centre+, Maastricht, the Netherlands; e Dutch Expert Centre for Gastrointestinal Ischaemia, Enschede, the Netherlands; f GROW - School for Oncology and Developmental Biology, Maastricht University Medical Centre+, Maastricht, the Netherlands

**Keywords:** Aortic dissection, Case report, Diagnosis, Intestinal fatty acid binding protein, Non-occlusive mesenteric ischaemia

## Abstract

**Introduction:**

Diagnosing non-occlusive mesenteric ischaemia (NOMI) in patients is complicated, due to poor signs and symptoms and non-specific laboratory tests, leading to a high mortality rate. This case study presents the rare case of a patient who developed mesenteric ischaemia after an emergency thoracic endovascular aneurysm repair (TEVAR) for a type B aortic dissection (TBAD) and peri-operative cardiogenic shock. Study outcomes revealed that intestinal fatty acid binding protein (I-FABP) identified early mucosal damage two days before the clinical presentation.

**Report:**

A 43 year old male patient was admitted to the emergency department with an acute TBAD and a dissection of the superior mesenteric artery (SMA), for which TEVAR was performed with additional stent placement in the SMA. Peri-operatively, the patient went into cardiogenic shock with a sustained period of hypotension. Post-operatively, the plasma I-FABP levels were measured prospectively, revealing an initial increase on post-operative day five (551.1 pg/mL), which continued beyond day six (610.3 pg/mL). On post-operative day seven, the patient developed a fever and demonstrated signs of peritonitis and bowel perforation. He underwent an emergency laparotomy, followed by an ileocaecal resection (<100 cm) with a transverse ileostomy. Pathological analysis confirmed the diagnosis of mesenteric ischaemia.

**Discussion:**

The diagnosis of NOMI in critically ill patients is often complicated, and the currently available diagnostic markers lack the specificity and sensitivity to detect early intestinal injury. This case report highlights that elevated I-FABP in plasma levels may indicate the presence of early mesenteric injury. Further research needs to be conducted before I-FABP can be applied in daily practice.

## Highlights

• Diagnosing non-occlusive mesenteric ischaemia (NOMI) is complicated due to the lack of specific and sensitive biomarkers.
• This report presents a unique case of NOMI secondary to a complicated acute type B aortic dissection.
• Elevated plasma intestinal fatty acid binding protein (I-FABP) levels may indicate the presence of early mesenteric injury.
• Further clinical research is required for the routine clinical application of plasma I-FABP.

## Introduction

Non-occlusive mesenteric ischaemia (NOMI) is a life threatening vascular emergency caused by diminished or interrupted blood flow to the intestine during low flow status.^1^^,^^2^ Diagnosing NOMI in critically ill patients is challenging due to its non-specific clinical presentation and the lack of an accurate diagnostic test.^3^ Early identification of NOMI is essential, as it has the potential to rapidly advance from reversible mucosal or mural damage to a subsequent irreversible phase characterised by transmural bowel damage and a high mortality rate.^1^^,^^2^ Ongoing studies are dedicated to uncovering biomarkers for NOMI, and intestinal fatty acid binding protein (I-FABP) is among the most promising candidates.^4^^,^^5^ I-FABP is a small (14 kDa) protein found in mature enterocytes at the tip of the villus. Earlier studies have already proven its functionality as a marker for enterocyte membrane integrity loss, a phenomenon characteristic of the early reversible stage of acute mesenteric ischaemia (AMI).^5^ A recent observational study of suspected NOMI in intensive care unit (ICU) patients found that there was a substantial increase in I-FABP concentrations among patients with intestinal necrosis compared with those without.^6^

This case study discusses the rare case of a patient who developed mesenteric ischaemia after a thoracic endovascular aneurysm repair (TEVAR) procedure for a type B aortic dissection (TBAD), continued into the superior mesenteric artery (SMA), and peri-operative cardiogenic shock. Prospective measurements of plasma I-FABP have shown promise as an early indicator of NOMI, as its elevation preceded the clinical presentation of NOMI by two days.

The patient was incapacitated during the screening process for inclusion in the study. As a result, written informed consent was obtained from his family for both study participation and the potential publication of future study results. After the patient regained consciousness, he was informed about the study and gave consent for participation. Any potentially identifiable images or data presented in this article have been appropriately anonymised to safeguard the patient's privacy.

## Report

**Figure 1:**
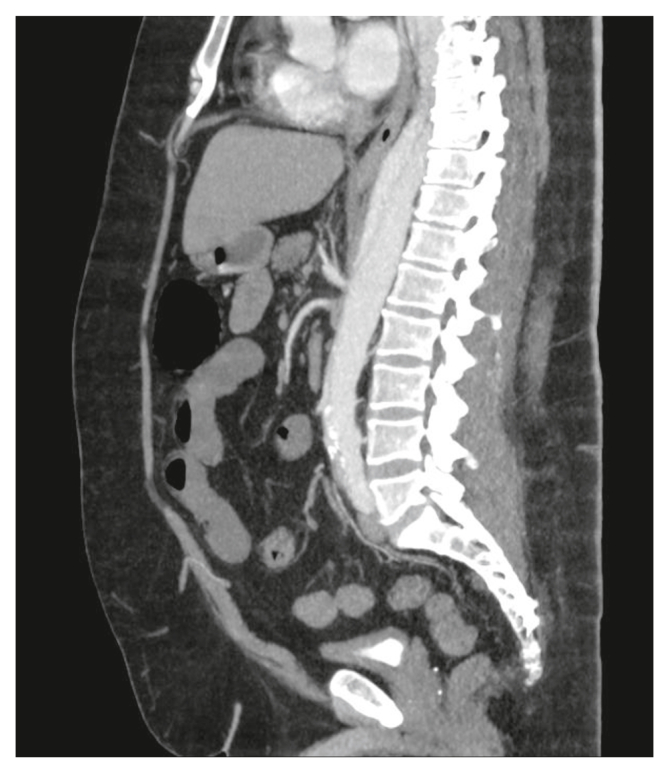
Abdominal computed tomography angiography showing a complicated type B aortic dissection of the thoracic and abdominal aorta and a dissection of the superior mesenteric artery with pre-existing atherosclerotic plaques.

A 43 year old obese male patient was admitted to the emergency department with severe abdominal pain, nausea, and abdominal tenderness in the right hemi-abdomen. Oesophagogastroduodenoscopy findings indicated a pale blue discoloration of the antrum, corpus, and duodenum with dark red discoloration of the intestinal mucosa with bulbitis and duodenitis. Computed tomography angiography (CTA) imaging demonstrated acute type B aortic dissection (TBAD) of the thoracic and abdominal aorta and a concomitant dissection of the SMA with pre-existing atherosclerotic plaques (Fig. 1 and Supplementary Videos A – C). The patient exhibited a clinical picture suggestive of acute gastrointestinal ischaemia with acute abdominal pain and nausea. Gastroduodenoscopy showed that there were indications of ischaemia in the stomach and duodenum.

Supplementary video related to this article can be found at https://doi.org/10.1016/j.ejvsvf.2024.04.004.

The following is/are the supplementary data related to this article:

Video S1 1

Video S2 2

Video S3 3

Multimedia component 5

The patient was transferred to the authors’ hospital and underwent an emergency TEVAR with stent placement in the SMA. Peri-operatively, he went into cardiogenic shock with a sustained period of hypotension and was placed on extracorporeal membrane oxygenation (ECMO), noradrenaline (Supplementary Fig), and continuous venovenous haemodialysis near the end of the procedure. The ECMO was discontinued on the third post-operative day. Accompanied by the presence of abdominal pain and leucocytosis upon admission, a clinical suspicion of NOMI was raised on the seventh post-operative day.

**Figure 2:**
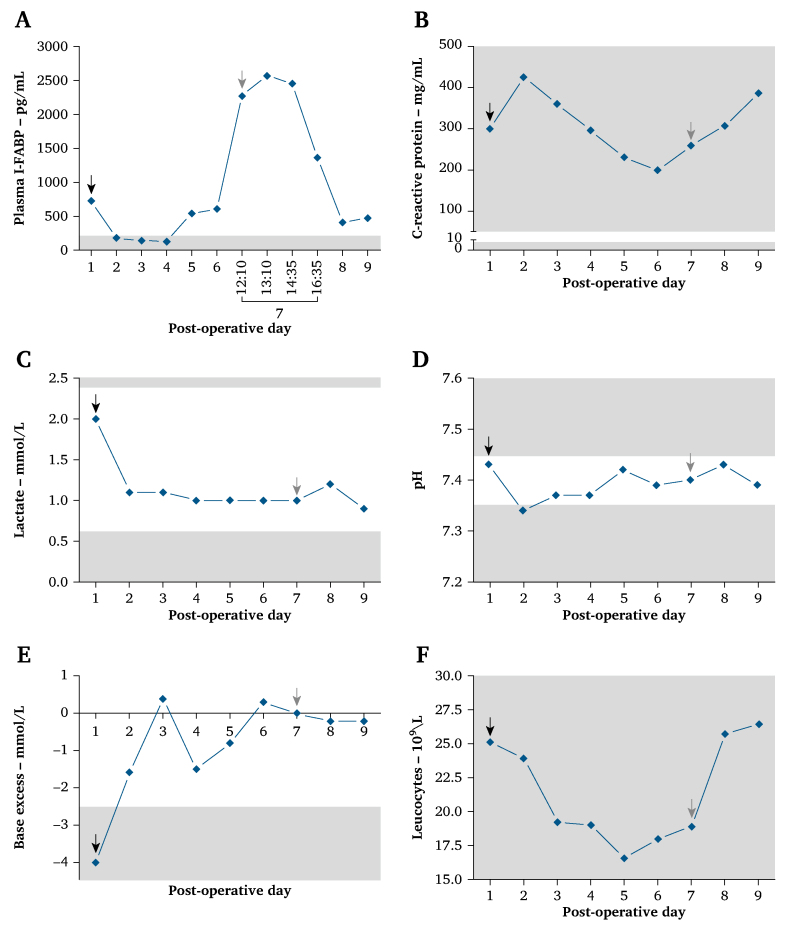
Graphs showing different laboratory parameters according to post-operative day. (A) Plasma I-FABP levels (pg/mL) were measured prospectively following the thoracic endovascular aneurysm repair (TEVAR) procedure, with post-operative blood plasma samples collected and analysed for up to nine days post-TEVAR. Measured laboratory parameters included: (B) C reactive protein, (C) lactate, (D) pH, (E) base excess, and (F) leucocytes. Post-TEVAR measurements are indicated by the black arrows, and grey arrows point to the emergency exploratory laparotomy followed by ileocaecal resection and ileostom. If a measured laboratory parameter falls within the white area (reference range), it is considered within normal limits. The upper and lower reference limits are illustrated in the graphs as the grey area. The mean plasma I-FABP levels in a group of healthy controls, set at 217.8 pg/mL, are portrayed in the highlighted grey area.^7^ Reference values for each parameter are: C reactive protein (<10 mg/L), lactate (0.5–1.7 mmol/L), pH (7.35–7.45), base excess (−2.5 – 2.5 mmol/L), and leucocytes (3.5–11.0 10^9^/L). All data were collected prospectively.

**Figure 3:**
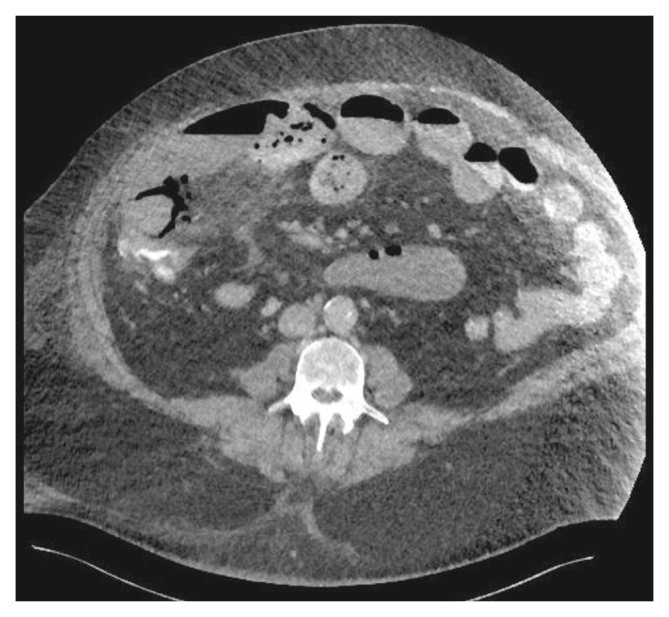
Abdominal computed tomography angiography of the patient revealed a distended bowel with pneumatosis intestinalis. Focally, the small intestine lacked coloration and showed infiltration of mesenteric fat tissue.

Prospective laboratory measurements were performed for I-FABP plasma levels (Fig. 2) as part of the TACTIC study.^5^ Following the TEVAR procedure, plasma I-FABP levels ranged 521.65–731.1 pg/mL. These levels rapidly decreased on the first day after the TEVAR procedure, reaching values consistent with those observed in healthy controls measured with the same assay;^7^ these lower I-FABP levels were sustained for the next two days. On the fifth post-operative day, the I-FABP plasma concentrations rose again (551.1 pg/mL) and persisted, reaching 610.3 pg/mL on the sixth post-operative day. Antibiotic treatment was initiated on day seven, when the patient developed a septic profile. A CTA scan showed a patent SMA stent and the presence of free intra-abdominal air and fluid on the right side of the abdomen with secondary signs of AMI, such as pneumatosis intestinalis and an indurated ileocaecal mesentery (Fig. 3). Prospective measurements also found an increase in plasma I-FABP levels (2271.5 pg/mL). An emergency exploratory laparotomy demonstrated faecal contamination in the right hemi-abdomen and multiple necrotic patches throughout the ileum. Approximately 100 cm of necrotic ileum together with the caecum was resected, followed by an ileostomy. Pathological analysis confirmed the diagnosis of transmural mesenteric ischaemia.

Post-operative laboratory measurements showed a decrease in plasma I-FABP levels (post-operative days 8–9). Following the laparotomy, the patient's condition remained stable throughout the post-operative period, aside from minor bleeding noted around the ileostomy and a positive blood culture. Therefore, antibiotic treatment was continued and adjusted based on blood culture findings. The patient spent 36 days in the ICU before being discharged after 46 days of hospitalisation and transferred to a rehabilitation clinic.

## Discussion

Non-occlusive mesenteric ischaemia is a rare abdominal emergency requiring rapid diagnosis, yet it is frequently missed due to its non-specific clinical presentation resulting in a high mortality rate.^1^^,^^2^ Existing diagnostic laboratory parameters used for NOMI diagnosis do not provide the necessary accuracy to comprehensively confirm both the early onset and, notably, the degree of intestinal tissue damage.^3^ Hence, a highly accurate, minimally invasive, and rapid diagnostic test is needed to confirm the NOMI diagnosis and initiate timely treatment.^8^

Non-occlusive mesenteric ischaemia often occurs as a consequence of a severe decrease in blood flow through the SMA. When the decrease in blood flow reaches approximately 50%, the splanchnic circulation rapidly responds with compensatory vasodilation.^9^ However, after several hours of low SMA flow this compensation becomes ineffective, leading to mesenteric vasoconstriction and a progressive increase in arterial resistance. If splanchnic vasoconstriction persists for >30 minutes, it becomes irreversible, even if full blood flowthrough the SMA is restored.

During the peri-operative period, this patient went into cardiogenic shock, marked by a sustained period of hypotension and elevation of lactate levels. These data suggest that cardiogenic shock during the procedure probably triggered the development of irreversible mesenteric vasoconstriction, ultimately leading to NOMI.^9^ The incidence of NOMI following acute major vascular surgery is not uncommon. A retrospective observational study investigating risk factors for NOMI following surgery revealed that the diagnosis is typically established after a mean of 8.1  ±  9.6 days post-surgery,^10^ while another study reported a median duration between cardiac surgery and a NOMI diagnosis of 14.0 (10.3–20.3) days.^11^ Despite advances in finding the pathophysiological mechanisms of NOMI, a comprehensive understanding remains elusive. Importantly, the prognosis and survival outcomes for patients predominantly rely upon early detection and therapeutic intervention.

A noticeable elevation in plasma I-FABP levels was detected two days prior to the clinical deterioration induced by transmural intestinal necrosis. Previous clinical studies have established a correlation between increased I-FABP levels and the early stages of intestinal ischaemia.^5^ A study involving ICU patients suspected of NOMI (DIAGOMI study) explored the diagnostic accuracy of I-FABP.^6^ Plasma I-FABP levels were significantly elevated in instances of intestinal necrosis, with an area under the ROC curve (AUC) of 0.83 (0.70–0.96). Furthermore, they determined that an I-FABP threshold of 3114 pg/mL had a sensitivity of 70% (50–86), specificity of 85% (55–98), negative predictive value of 58% (36–93), positive predictive value of 90% (67–96), positive likelihood ratio of 4.57 (1.25–16.75), and negative likelihood ratio of 0.35 (0.19–0.65) for diagnosing intestinal necrosis. The I-FABP measurements obtained in this case study were lower (pre-operative, 2568.37 pg/mL) compared with the findings in the DIAGOMI study.^6^ In addition, the I-FABP reference level shown in Fig. 3 was based on the mean I-FABP plasma levels in healthy individuals,^7^ whereas the I-FABP threshold from the DIAGOMI study aimed to identify cases of intestinal necrosis. There is currently no consensus on a clinical I-FABP threshold for intestinal ischaemia,^3^^,^^8^ and the plasma I-FABP levels vary depending on the specific ELISA assay;^12^ therefore, future studies are needed to explore the efficacy of I-FABP (possibly in combination with other biomarkers) in larger patient cohorts and across different types of AMI to establish an accurate diagnostic threshold for future clinical use.^8^ Research endeavours are currently underway to establish I-FABP thresholds and investigate alternative biomarkers for mesenteric ischaemia. The Detrimental Course of Acute Intestinal Ischaemia (TACTIC) study is one example, as it is examining a panel of plasma biomarkers, including I-FABP, with the goal of enabling early and precise identification of acute intestinal ischaemia in patients.^13^ The TACTIC study is expected to yield results in 2024.

To conclude, this case study presents an unique case involving a patient who developed NOMI following major vascular surgery and secondary peri-operative cardiogenic shock in whom I-FABP plasma levels predicted NOMI two days before its clinical manifestation. Further research needs to be conducted to elaborate on I-FABP functionality in different types of AMI and validate these findings against current clinical diagnostic standards.

## Conflicts of interest

None declared.

## Funding

This work was supported by the Dutch Digestive Foundation (Maag Lever Darm Stichting, MLDS), Amersfoort, The Netherlands (grant number D17-14).

## Ethics approval

The (TACTIC) study protocol was approved (4 September 2019) and registered by the ethical review board of the Maastricht University Medical Centre+ (MUMC+) in Maastricht, The Netherlands (METC19-010) and at ClinicalTrials.gov (NCT05194527 or https://clinicaltrials.gov/ct2/show/NCT05194527).^13^

## Availability of data and material

The authors confirm that the data supporting the findings of this study are available within the article. In addition, raw data supporting these findings results are available from the corresponding author upon reasonable request. Data were visualised using GraphPad Prism 8 software.
