# Supplementary material for: Intestinal Fatty Acid Binding Protein as a Predictor of Early Mesenteric Injury Preceding Clinical Presentation: A Case Report
Source: EJVES Vasc Forum. 2024 Apr 29;61:136–40. doi: 10.1016/j.ejvsvf.2024.04.004 (PMC11176664; doi:10.1016/j.ejvsvf.2024.04.004)
Supplement: Multimedia component 5 [file mmc5.docx]

**Supplementary Video A – C.** Contrast-enhanced computed tomography (CT) scans were acquired in the (A) frontal, (B) sagittal, and (C) transverse planes. The CT imaging revealed a complicated type B-aortic dissection of the thoracic and abdominal aorta, and a dissection of the superior mesenteric artery with pre-existing atherosclerotic lesions. The dissection extended into both the left common iliac artery and the right external iliac artery, primarily receiving its blood supply from the false lumen. The truncus celiacus and the arteria mesenterica inferior arose from the true lumen. Variable enhancement of the intestinal walls was observed, with focal increased enhancement noted, particularly in the region of the small intestine, without signs of transmural ischaemia.
