# Supplementary material for: Intestinal Fatty Acid Binding Protein as a Predictor of Early Mesenteric Injury Preceding Clinical Presentation: A Case Report
Source: EJVES Vasc Forum. 2024 Apr 29;61:136–40. doi: 10.1016/j.ejvsvf.2024.04.004 (PMC11176664; doi:10.1016/j.ejvsvf.2024.04.004)
Supplement: Multimedia component 1 [file mmc1.docx]

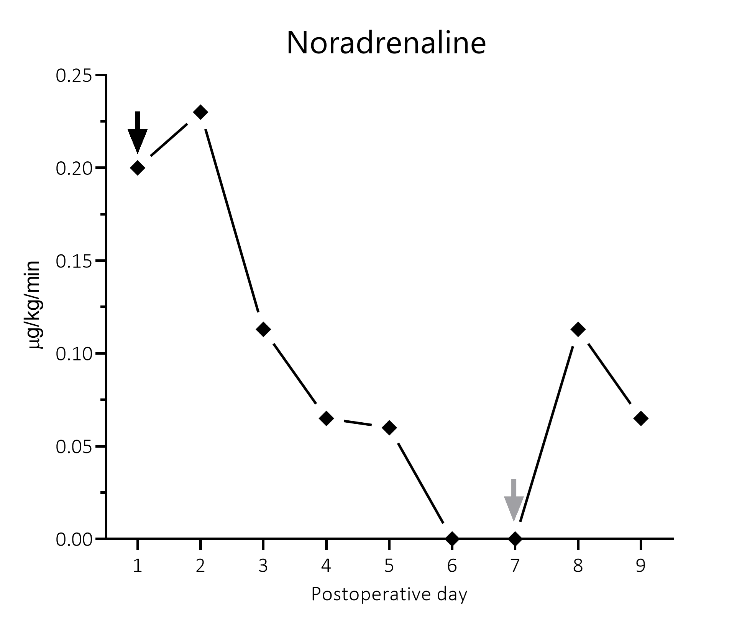


**Supplementary Figure.** The patient underwent peri-operative ECMO placement (black arrow), remaining on ECMO until post-operative day 3. Additionally, post-operatively, the patient received vasopressors (noradrenaline/norepinephrine) with an initial dose of µg/kg/min. The dosage was elevated on day 2 post-operatively (0.23 µg/kg/min). Subsequently, the noradrenaline dosage was gradually decreased between post-operative days 2 – 6. Following the emergency laparotomy (grey arrow), the patient was reintroduced to noradrenaline (0.113 µg/kg/min), with further dosage reduction in the subsequent days.
